# Rebuttal to "Life-threatening cardiac tamponade: a rare complication of acupuncture": Who framed acupuncture?

**DOI:** 10.1186/s13019-014-0144-2

**Published:** 2014-08-28

**Authors:** Tae-Hun Kim, Jung Won Kang

**Affiliations:** College of Oriental medicine, Gachon University, Seongnam, South Korea; Department of Acupuncture & Moxibustion, College of Korean Medicine, Kyung Hee University, Seoul, South Korea

**Keywords:** Acupuncture, Cardiac tamponade, Possible causes, Cardiopulmonary resuscitation, Chemotherapy, STRICTA

## Abstract

A rebuttal to Chun KJ, Lee SG, Son BS, Kim Do H: **Life-threatening cardiac tamponade: a rare complication of acupuncture**. *J Cardiothorac Surg* 2014, **9:** 61.

## Letters to the editor

Chun et al. reported a rare case of the cardiac tamponade which might be induced by acupuncture in the chest [1]. A middle aged woman was brought to the authors' hospital after cardiopulmonary resuscitation CPR. She had low systolic blood pressure and rapid heart rate when arrived. Before transferred to the hospital, she presented bradycardia, syncope and comatose mental state after treated with 30 mm-length acupuncture needle in left parasternal area of 4th intercostal space. She was diagnosed to be traumatic hemopericardium and recovered through surgery.

Before discussing the causality relation between acupuncture and cardiac tamponade, we firstly infer the possible causes around this life-threatening accident based on the primary reporting of this case [1]. If we look into this case carefully, there were many potential factors which could be the major cause of cardiac tamponade. This patient had participated in chemotherapy after mastectomy several years ago. According to a recent review, cardiac tamponade can spontaneously occur among cancer patients who had chemotherapy previously [2]. In addition, before arriving at the hospital, she had taken CPR due to bradycardia, syncope and comatose mental state, where we are quite curious whether these symptoms are sufficient indication for CPR because those symptoms seem to be more closely related to faint during acupuncture treatment, a strong symptom related to vagal reflex [3], rather than to cardiac arrest. Anyway, there are several studies that CPR can induce cardiac tamponade [2],[4]. Considering presentation of the case, acupuncture in the chest might be one of the potential candidates for the direct cause of cardiac tamponade. In this sense, it will be necessary to check all the possible causes to identify direct cause of cardiac temponade cautiously when dealing with this case: we need to consider the background of patient’s condition.

Another suspicious point is size of the injury. The patient had a perforating hole with 3 mm in diameter which the authors insisted the fatal legion made by acupuncture. But general types of acupuncture needles used by doctors of Korean Medicine have 0.25 mm in diameter and the thickness does not exceed 0.4 mm at best. To make a 3 mm hole with a needle of 0.25 mm in diameter looks unreasonable. From the above cases of CPR-related cardiac tamponade, size of the injuries to the heart is similar to that of the case presented in this report, if anything [2],[4].

We also have a suspect point about the acupuncture treatment itself in this case. The acupuncture point, when deduced by the original report, seems to be KI23 (Sinbong) which is selected for angina, mastalgia, dyspnea and intercostal pain according to the Korean textbook of classic acupuncture [5]. Although there are several indications suggested in the textbook, however, clinicians do not prefer to use this point because they know that the point is very dangerous but the therapeutic effect cannot be assured: harm is expected to surpass benefit. Direction and depth are taught to be decided carefully when acupuncture needles are inserted at anterior, lateral and posterior chest wall in classic acupuncture [6]. Acupuncture points located in the anterior chest wall are not selected frequently, and when acupuncture treatment is necessary, only 6.6 to 9.9 mm are recommended to avoid acupuncture-related adverse effects. There is no physician in Korea who has been appropriately educated and inserts acupuncture needle at the acupuncture points located in the chest with 30 mm-depth. In this aspect, the pattern of acupuncture of this case report cannot be accepted as a common practice from the expert’s view.

Apart from these odd things from the clinical context, there seem to be several issues related to the manner of reporting. Acupuncture is a complex intervention with wide variety in its clinical usage. In this sense, components in details need to be declared for better understanding of clinical situation when reporting acupuncture treatment. STRICTA checklist is recommended which contains necessary items for identifying exact types of acupuncture [7]. Acupuncture rationale about the principle of acupuncture treatment, details of needling including acupuncture points, depth and direction of needling, stimulating methods, features of acupuncture needle and practitioner’s background including degree of clinical expertise are important information for reasoning whether this event can occur by a normal acupuncture practice or occur by a malpractice. We already pointed this out in a different report on the adverse events related to acupuncture [8]. What educational basis and how long clinical career does the professional acupuncturist have as the authors referred to the person who might conduct acupuncture? Why did he/she select acupuncture points in the anterior chest wall which are not treated in usual practice? Without these information, the value of this case report can be degraded to the slander of exaggerating harm of acupuncture treatment.

## Abbreviations

CPR: cardiopulmonary resuscitation
STRICTA: STandards for Reporting Interventions in Clinical Trials of Acupuncture
